# Aseptic Vaccination in New York

**Published:** 1899-09-30

**Authors:** 


### ASEPTIC VACCINATION IN NEW YORK.

We may fairly believe that the Vaccination Act and the order on the subject issued by the Local Government Board have done good to at least one branch of the community, namely, the surgical instrument makers, for no vaccinator nowadays seems to regard his outfit as complete unless he carries with him some patent arrangement for sterilising his instruments. In America they get over the sterilisation difficulty in a somewhat different manner. In New York there has lately been some anxiety about small-pox, and it has been determined that the school law concerning vaccination shall be strictly enforced, and we are told by the *Medical News* that all precautions are taken by the physician of the health board in the performance of the vaccination. "In every case a new needle," we are told, "is used to scratch the arm, and by means of a fresh, unused splinter of wood the virus is spread over the wound."

